# Anaemia, Haemoglobin Level and Cause-Specific Mortality in People with and without Diabetes

**DOI:** 10.1371/journal.pone.0041875

**Published:** 2012-08-02

**Authors:** Andre Pascal Kengne, Sébastien Czernichow, Mark Hamer, G. David Batty, Emmanuel Stamatakis

**Affiliations:** 1 National Collaborative Research Programme on Cardiovascular and Metabolic Disease, South African Medical Research Council and University of Cape Town, Cape Town, South Africa; 2 Julius Center for Health Sciences and Primary Care, University Medical Center Utrecht, Utrecht, The Netherlands; 3 Cardiovascular Division, The George Institute for Global Health, Sydney, Australia; 4 Department of Nutrition, Ambroise Paré Hospital (AP-HP), Boulogne-Billancourt, France; 5 Department of Nutrition, University of Versailles St-Quentin, Boulogne-Billancourt, France; 6 Department of Epidemiology and Public Health, University College London, London, United Kingdom; Innsbruck Medical University, Austria

## Abstract

**Background:**

Both anaemia and cardiovascular disease (CVD) are common in people with diabetes. While individually both characteristics are known to raise mortality risk, their combined influence has yet to be quantified. In this pooling project, we examined the combined impact of baseline haemoglobin levels and existing CVD on all-cause and CVD mortality in people with diabetes. We draw comparison of these effects with those apparent in diabetes-free individuals.

**Methods/Principal Findings:**

A combined analyses of 7 UK population-based cohorts resulted in 26,480 study members. There were 946 participants with physician-diagnosed diabetes, 2227 with anaemia [haemoglobin<13 g/dl (men) or <12 (women)], 2592 with existing CVD (stroke, ischaemic heart disease), and 21,396 with none of the conditions. Across diabetes and anaemia subgroups, and using diabetes-free, non-anaemic participants as the referent group, the adjusted hazard ratios (HR) were 1.46 (95% CI: 1.30–1.63) for anaemia, 1.67 (1.45–1.92) for diabetes, and 2.10 (1.55–2.85) for diabetes and anaemia combined. Across combined diabetes, anaemia and CVD subgroups, and compared with non-anaemic, diabetes-free and CVD-free participants, HR (95% CI) for all-cause mortality were 1.49 (1.32–1.69) anaemia, 1.60 (1.46–1.76) for existing CVD, and 1.66 (1.39–1.97) for diabetes alone. Equivalents were 2.13 (1.48–3.07) for anaemia and diabetes, 2.68 (2.14–3.36) for diabetes and existing CVD, and 3.25 (1.88–5.62) for the three combined. Patterns were similar for CVD mortality.

**Conclusions/Significance:**

Individually, anaemia and CVD confer similar mortality risks in people with diabetes, and are excessively fatal in combination. Screening for anaemia would identify vulnerable diabetic patients whose outcomes can potentially be improved.

## Introduction

Anaemia is frequent in people with diabetes where it is generally undetected and therefore untreated [1], [2]. There is evidence that anaemia is associated with microvascular complications of diabetes [2], [3], [4]. In both diabetic and nondiabetic subjects, anaemia is a determinant of all-cause and cardiovascular disease (CVD) mortality [1], [5], [6]; although whether such association is consistent across a broader population with diabetes is still equivocal [7], [8], [9].

CVD is common in people with diabetes [10] and CVD often co-exists with anaemia. While individually, both CVD and anaemia are known to raise mortality risk, their combined influence has yet to be quantified, particularly in the general population. Accordingly, in this pooling project, we examined the combined association of baseline haemoglobin levels and existing CVD with all-cause and CVD mortality in people with diabetes. We draw comparison of these effects with those in people who are diabetes-free.

## Materials and Methods

Participants were 26,480 individuals with data available on diabetes status (history of physician-diagnosed) and total haemoglobin level at baseline [11]. Study members were drawn from 7 prospective UK studies comprising both Scottish Health Surveys (1995 & 1998) and the Health Surveys for England (1994, 1998, 1999, 2000 & 2004) [11], [12]. All cohorts were representative of the general population, sampling individuals living in households in each country. Participants gave full informed written consent and ethical approval was obtained from the London Research Ethics Council.

The full study protocol has been described in detail elsewhere [13], [14], [15]. In brief, participants were visited twice in their homes. During the first of these meetings, trained interviewers collected data on demographics and health behaviours, including socioeconomic status, self-reported smoking, alcohol and physical activity. Interviewers made enquiries about existing physician-diagnosed CVD (stroke, ischemic heart disease, angina symptoms), other medical conditions and treatments. During the second visit, conducted within a few days of the first, nurses gathered clinical data. This included total haemoglobin which was assayed from a non-fasting peripheral blood sample.

Systolic and diastolic blood pressure was measured with an Omron HEM-907 blood pressure monitor three times in the sitting position after 5-min rest between each reading. The average of the second and third BP recordings was used for the present analyses. Height and weight were measured directly by the interviewers using Chasmors stadiometers (Chasmors Ltd, London, UK) and Tanita electronic digital scales (Tanita, Corporation, Tokyo, Japan), respectively. BMI was calculated using the usual formulae (weight [kg]/height [m2]). Waist and hip circumferences were measured using a tape with an insertion buckle at one end. Waist circumference was measured at the midpoint between the lower rib and the upper margin of the iliac crest. Hip circumference was denoted by the widest circumference around the buttocks, below the iliac crest. Both measurements were taken twice, using the same tape, and were recorded to the nearest even millimetre. Those whose two waist or hip measurements differed by more than 3 cm had a third measurement taken. The mean of the two valid measurements was used in our analysis. Cholesterol was measured using cholesterol oxidase assays on an Olympus 640 analyzer. Anaemia was defined as haemoglobin concentrations <13 g/dl (men) and <12 g/dl (women), following the World Health Organisation criteria [16].

### Ascertainment of Disease-specific Mortality

Consenting participants were linked to UK National Health Service records from which a death certificate was located. Classification of the underlying cause of death was based on information on the death certificate together with any additional observations made by the certifying physician. Diagnoses for primary cause of death used the ninth (ICD-9) and tenth (ICD-10) revisions of the International Classification of Diseases. Cardiovascular disease codes were 390–459 for ICD-9 and I01–I99 for ICD-10.

### Statistical Methods

The starting study sample comprised 57,073 participants, among whom 28,809 (46.2%) had provided blood sample for total haemoglobin assays. Sixteen were excluded for missing data on diabetes status. Other 2313 participants who did not consent for mortality follow-up were also excluded. Therefore primary analyses were based on 26,480 individuals (12,135 men) with data available on age, sex, diabetes status and haemoglobin level at baseline (Figure S1). Of these participants, 23,129 had complete data on covariates and were included in multivariable model analyses. In the Table S1 we present the baseline characteristics of study members included and excluded from the analytical sample. Differences were small in magnitude and clinically trivial but attained statistical significance for many characteristics owing to the large numbers. For instance, mean baseline variables (participants in the primary analysis vs. those excluded) were 26.9 vs. 27.3 kg/m^2^ for body mass index, 90 vs. 91 cm for waist circumference, 0.86 vs. 0.87 for waist/hip ratio and 5.9 vs. 5.8 mmol/l for total cholesterol (all p≤0.002 for difference).

Participants were classified according anemia and prior CVD status, then further grouped by baseline diabetes status. Baseline comparisons used logistic regressions and generalized linear regression models. A Poisson model was used to determine the absolute risk of CVD and all-cause mortality during follow-up by status for anaemia, and for prior CVD. Kaplan-Meier estimator was used to compute the probability of death during follow-up and estimates compared across baseline stratification variables with the use of the Log-Rank test. Cox regression models were used to investigate the associations of anaemia and prior CVD with mortality after adjustment for cohort, age, sex, smoking, systolic blood pressure, body mass index and total cholesterol.

To investigate the association between total haemoglobin and mortality risks, Cox models were used to compute the hazard ratio and accompanying 95% confidence interval (95% CI) for a one standard deviation (SD) decrease in total haemoglobin in relation to all-cause and CVD mortality. Similar Cox models were used to compare mortality risk across fifths of haemoglobin, with 95% CI derived with the used of floating absolute risk methods [17]. Fifths of haemoglobin were sex specific to account for sex differences in the distribution of haemoglobin. The ‘shape’ of the associations of haemoglobin with mortality risks was investigated with the use of restricted cubic spline and by fitting the polynomial terms of haemoglobin. Different functional forms were compared through likelihood ratio χ^2^ and Akaike’s information criterion (AIC). [18] Data analyses used SAS/STAT® v 9.1 for windows (SAS Institute Inc., Cary, NC, USA) and the statistical package R v.2.12.2 [(2011-02-25), The R Foundation for statistical computing, Vienna, Austria].

### Sensitivity Analyses

Among eligible participants, 13,228 (424 with diabetes, 3.3%) had data available on CRP levels. Multivariable Cox regression analyses were conducted in this subgroup with further adjustment for CRP levels to investigate the potential effects of chronic inflammation on the observed results.

## Results

### Baseline Profile

The study sample included 26,480 participants among whom 946 (3.6%) had diabetes. The prevalence of anaemia was higher (14.3%; n = 135) in participants with diabetes than those without (8.2%; n = 2092; p-value for difference <0.001). Compared to their non-anaemic counterparts, participants with anaemia were more likely to be female, to smoke, and have with a history of CVD. They were also older but had lower systolic blood pressure, body mass index, waist circumference and total cholesterol (Table 1). In addition, the expected adverse profile of risk factors was observed in participants with diabetes as compared to those without. The characteristics of Participants cross-classified by anaemia and prior CVD are presented in Table S2. Among diabetic and nondiabetic participants, those with anaemia and existing CVD were older and less likely to be current smokers (all p≤0.008) Differences were also apparent in the distribution of other baseline variables, however with no consistent pattern (Table S2). The characteristics of participants according to status for diabetes and across fifths of total haemoglobin distribution are shown in Table S3. Age decreased with increasing haemoglobin, while increasing trend was observed for other variables (all p≤0.02 for linear trend).

**Table 1:** Baseline characteristics by status for anaemia and diabetes.

Variables	No diabetes			Diabetes			All participants	
	no anaemia	Anaemia	p-value	no anaemia	anaemia	p-value	Anaemia (yes vs. no)	Diabetes (yes vs. no)
N	23442	2092		811	135			
Women (%)	53.1%	70%	<0.001	44%	49.6%	0.22	<0.001	<0.001
Mean age, years (SD)	54.4 (13.2)	59.0 (17.1)	<0.001	61.9 (11.9)	68.8 (14.2)	<0.001	<0.001	<0.001
Current smoking (%)	27.4%	15.2%	<0.001	24.3%	11.9%	0.001	<0.001	0.008
Mean systolic bloodpressure*, mmHg (SD)	137 (20)	134 (22)	<0.001	146 (21)	145 (25)	0.57	<0.001	<0.001
Mean resting heart rate,bpm^†^ (SD)	71 (11)	71 (11)	0.13	74 (12)	74 (10)	0.65	0.06	<0.001
Mean body mass index,kg/m^2^ (SD)	27.0 (4.5)	25.8 (4.8)	<0.001	29.1 (5.1)	26.8 (4.1)	<0.001	<0.001	<0.001
Mean waist circumference,cm (SD)	90.0 (12.9)	85.7 (12.4)	<0.001	98.6 (13.1)	94.1 (11)	<0.001	<0.001	<0.001
Mean waist/hip ratio (SD)	0.86 (0.09)	0.84 (0.08)	<0.001	0.92 (0.08)	0.91 (0.07)	0.10	<0.001	<0.001
Mean total cholesterol,mmol/l (SD)	6.0 (1.2)	5.5 (1.1)	<0.001	5.8 (1.1)	5.3 (1.2)	<0.001	<0.001	<0.001
Median CRP, mg/l (25^th^–75^th^ percentiles)	1.8 (0.8–3.9)	1.6 (0.6–4.6)	<0.001	3.1 (1.5–6.4)	3.5 (1.0–16.6)	0.007	<0.001	<0.001
Existing cardiovasculardisease (%)	8.7%	14.3%	<0.001	26.6%	31.1%	0.28	<0.001	<0.000

SD, standard deviation.

### Effects of Anaemia on Mortality Risk

During follow-up, 4643 deaths from all cause were recorded, of which 1347 (29%) were from cardiovascular disease. The number of fatal outcomes in participants with diabetes was 378 for all-cause mortality and 133 for cardiovascular mortality.

The absolute risk (95% CI) per 1000 person-years for participants with and without diabetes, and by status for anaemia at baseline is shown in Table 2. In both people with and without diabetes, anaemia was associated with increased incidence of all-cause and CVD mortality. Absolute risk of fatal events in nondiabetic participants with anaemia was always lower than that in diabetic participants without anaemia (Table 2).

**Table 2:** Incidence all-cause and Cardiovascular and all-cause mortality per 1000 person-years of follow-up and hazard ratios.

Baseline classification		CVD mortality			All-cause mortality		
Diabetes	Anaemia	Event rate(/1000 pys)	HR (95% CI)*	HR (95% CI)†	Event rate(/1000 pys)	HR (95% CI)*	HR (95% CI)†
No	No	4.2 (4.0–4.5)	1	1	14.8 (14.4–15.3)	1	1
No	Yes	9.3 (8.7–11.7)	1.48 (1.20–1.81)	1.53 (1.24–1.88)	33.5 (31.0–36-2)	1.44 (1.29–1.60)	1.46 (1.30–1.63)
Yes	No	15.2 (12. 6–18.3)	2.32 (1.84–2.94)	2.00 (1.57–2.53)	41.8 (37.3–46.9)	1.76 (1.53–2.02)	1.67 (1.45–1.92)
Yes	Yes	29.0 (19.6–42.9)	2.08 (1.17–3.70)	1.96 (1.10–3.50)	90.5 (72.5–113.0)	2.22 (1.64–3.01)	2.10 (1.55–2.85)

* Cox models are adjusted for cohort, age, sex,

† Cox models are further adjusted for smoking systolic blood pressure, total cholesterol, BMI and prior CVD.

CI, confidence interval; HR, hazard ratio; prs, person-years.

**Figure 1:**
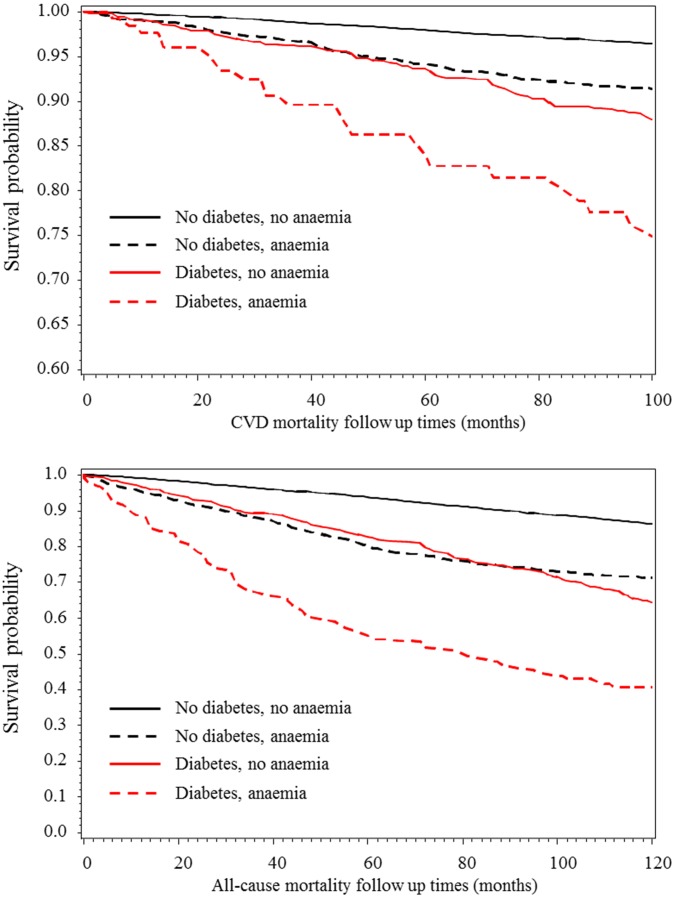
Kaplan-Meier estimates of the probability of all-cause and cardiovascular mortality during follow-up in participants with and without diabetes, and by status for anaemia. The upper figure panel is for cardiovascular disease and the lower for all-cause mortality.

The probability of survival during follow-up is depicted in Figure 1. At any given time, the highest probability was always recorded in nondiabetic non-anaemic participants and the lowest in diabetic participants with anaemia. Across diabetes and anaemia strata, survival probabilities among diabetes-free participants with anaemia were similar to those among diabetic participants without anaemia (Figure 1).

Using nondiabetics without anaemia as a reference group, anaemia without diabetes was associated with 53% (24–88%) higher risk of CVD mortality after adjustment for age, sex, prior CVD, systolic blood pressure, current smoking, body mass index and total cholesterol. Equivalents were 100% (57–153%) for diabetes without anaemia and 96% (10–250%) for diabetes & anaemia (Table 2).

### Combined Effects of Anaemia and Existing CVD on Morality Risk

**Figure 2:**
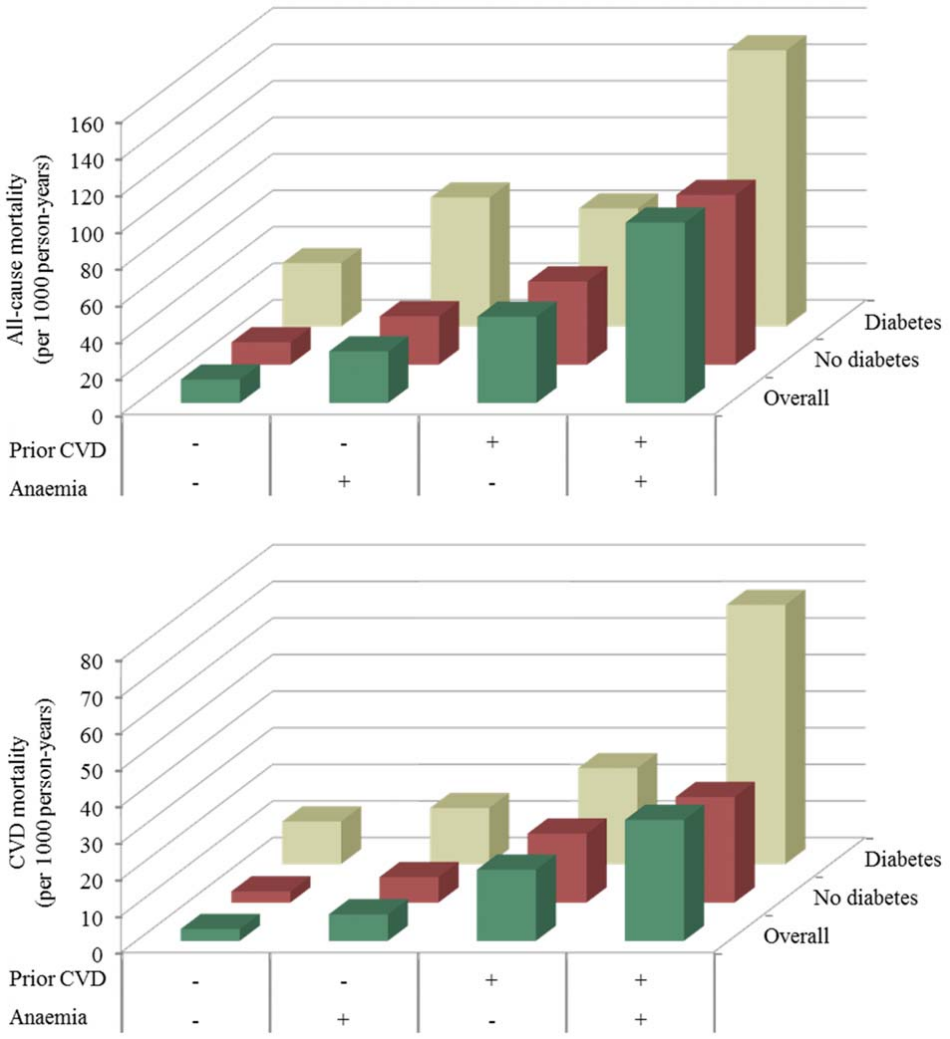
Incident all-cause and cardiovascular disease (CVD) mortality (per 1000 person-years of follow-up) in participants with and without diabetes, with further stratification by status for anaemia and existing CVD. + denotes the presence of the characteristic, and – denotes its absence.

The absolute risk of all-cause and CVD mortality (per 1000 person-years) by crossed status for anaemia and existing CVD is summarised in Figure 2. For each grouping category by both anaemia and existing CVD status, absolute risks were always higher in diabetic than in non-diabetic participants. Absolute risks were within the same range among diabetes-free participants with anaemia and existing CVD, non-anaemic diabetic participants with existing CVD, and anaemic diabetic participants with no prior CVD. Having diabetes, anaemia and existing CVD at least doubled the risk from having only any two combinations of the three. These similarities and differences were consistent through follow-up and after adjustment for several baseline characteristics (Table S4). For instance, using nondiabetic participants without anaemia and no existing CVD as a reference, anaemia alone was associated with hazard ratio (95% CI) of 1.49 (1.32–1.69) for all-cause mortality, while diabetes was associated with 1.66 (1.39–1.97). Equivalents were 2.13 (1.48–3.07) for anaemia and diabetes; 2.68 (2.14–3.36) for diabetes and existing CVD; and 2.14 (1.73–2.66) for anaemia and existing CVD.

### The Continuum of Total Haemoglobin and Mortality

**Figure 3:**
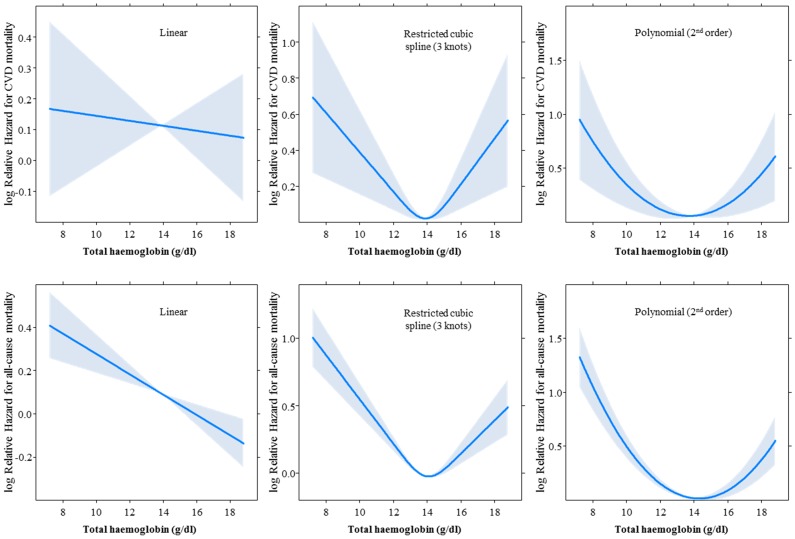
Effect of various coding of total haemoglobin on the association with cardiovascular disease (upper panels) and all-cause (lower panels) mortality in age, sex and cohort adjusted Cox regression models. The solid curve depicts the shape of the shape of the association across the continuum of total haemoglobin, and the shaded area if for the 95% confidence interval around the curve.

In age, sex and cohort adjusted analysis, there was a weak positive association between haemoglobin and all-cause mortality [hazard ratio per SD lower haemoglobin: 1.07 (95% CI: 1.03–1.10), with no significant heterogeneity by diabetes status (p = 0.08 for interaction). No continuous association was found for CVD mortality. The shape of the associations for different coding of total haemoglobin is depicted in Figure 3, and accompanying fits statistics in Table S5. The linear form was always the least fitting functional form, and curvilinear forms (U-shape) were always the best fitting, with nadir of risk around 14 g/dl for haemoglobin levels. Adjusted hazard ratios and confidence intervals for mortality risk across quintiles of haemoglobin as depicted in Figure S2. Using the top quintile as reference, significant higher risk of mortality was observed only within the lowest quintile.

A total of 64 participants (5 with diabetes) had haemoglobin levels within the range for defining polycythaemia (i.e. haemoglobin≥18.5 g/dl in men or haemoglobin≥16.5 g/dl). Among them 30 deaths (cumulative incidence 46.9%) were recorded, 5 (cumulative incidence 7.8%) being of cardiovascular origin. Using participants with normal range haemoglobin levels as a reference group, the age and sex adjusted hazard ratios (95% confidence intervals) associated with all-cause mortality were 1.46 (1.34–1.59) for anaemia and 2.70 (1.89–3.88) for polycythaemia. The equivalents for CVD mortality were 1.28 (1.10–1.50) and 1.56 (0.65–3.77).

### Sensitivity Analyses

Among participants with data available on CRP levels (13,228 participants), 1761 (136 in people with diabetes) deaths were recorded during follow-up, of which 590 (61 in people with diabetes) were from cardiovascular disease. Across diabetes and anaemia strata, using nondiabetics without anaemia as a reference group, the age and sex adjusted HR (95% CI) associated with CVD mortality were 1.55 (1.17–2.04) for anaemia without diabetes, 2.68 (2.01–3.58) for diabetes without anaemia and 2.23 (1.10–4.53) for both anaemia and diabetes. The equivalents after further adjustment for prior CVD, systolic blood pressure, current smoking, body mass index, total cholesterol and log(CRP) were 1.43 (1.08–1.90), 2.13 (1.58–2.86) and 1.81 (0.90–3.70). For all-cause mortality, estimates were 1.39 (1.18–1.64), 1.83 (1.51–2.21) and 2.20 (1.44–3.36) in sex age adjusted models, and 1.24 (1.05–1.47), 1.65 (1.36–2.01) and 1.76 (1.15–2.71) after adjusted for all covariates including CRP. The small number of participants in some subgroups hampered our ability to reliably perform similar analysis across diabetes, anaemia and prior CVD strata. Estimates however were mostly similar to those from the main analysis (Table S4).

## Discussion

In this pooling of contemporary, community-based cohort studies, we found that anaemia was associated with increased risks of all-cause and cardiovascular disease mortality. The magnitude of these risks in people with diabetes and no history of CVD were similar to those conferred by a history of CVD. In diabetic participants with existing CVD, anaemia conveyed very high mortality risk. There was no continuous linear association between haemoglobin levels and mortality risk. Both lower and higher haemoglobin levels were associated with higher mortality risks.

### Prior Studies

Previous reports have separately examined the effects of anaemia or existing CVD on the risk of major outcomes. Investigations on the effects of anaemia have mostly focused on people with chronic kidney disease and have largely confirmed the related high risk of mortality [1], [5]. Other reports have consistently shown excess mortality risk in the presence of a prior CVD, regardless of diabetes status [10], [19], [20]. Available studies on anaemia and mortality risk in nondiabetics with existing CVD have been inconsistent, with findings ranging from no association in one study [21] to significant adverse association in two others [22], [23]. There is no similar data for people with diabetes.

We found that in both people with and without diabetes, anaemia was associated with increased risk of mortality in participants with prior CVD. In accordance with our results, previous population-based studies found that the association between haemoglobin levels and cardiac events, if any, was not linear [24]. However, splines and polynomial models showed that excess mortality would occur more in relation with lower than higher haemoglobin levels.

### Mechanisms of Effects

Anaemia in diabetes has been attributed to erythropoietin (EPO) deficiency subsequent to renal complications [1]. There are suggestions however that it is more complex and multifactorial and also include inflammation, nutritional deficiencies, autoimmune disease, drugs and hormonal changes [25]. The normocytic normochromic nature of anaemia, and the inverse relationship between haematocrit and C-reative protein levels in diabetes support the possible role of inflammation [26]. Haemoglobin concentrations are inversely related with glycated albumin [26] and hyperglycaemia is possibly associated with decreased erythrocytes lifespan [27], indicating a likely contribution of glucose control to anaemia.

### Anaemia Correction and Mortality Risk

Trials of anaemia correction to reduce cardiovascular risk conducted so far, exclusively in people with CKD, support a harmful effect of anaemia correction particularly when erythropoiesis-stimulating agents are used to raise haemoglobin levels into ‘normal range’ [28], [29], [30]. Whether those adverse outcomes were the effects of achieved haemoglobin, or strategies for correcting anaemia, have not been elucidated [31]. In general, available results should be interpreted with caution, with suggestions however that, for the time being, aggressive normalisation of haemoglobin levels in CKD patients with anaemia should be avoided. Evidence from others field suggests that anaemia correction using erythropoiesis stimulating agents is also harmful in patients with cancers [32], but not in those with heart failure [33]. However, caution is needed when extrapolating findings from trials in CKD patients to a broader population with may be less severe anaemia [7].

### Limitations and Strengths

Our study has some limitations. We lacked data on kidney function and could not account for possible effects of nephropathy [8]. Some studies [8], but not all [7], have found interactions between anaemia and presence of CKD for mortality risk, and were modulated by diabetes status and existence of a prior CVD. The current analyses were based on physician diagnosis. Therefore some participants with undiagnosed diabetes would have been misclassified as nondiabetics. Strengths of this study include the large number of participants, randomly selected from the general population, and consistency of survey methods across included cohorts, making our findings generalizable to broader populations. Unlike most previous studies, we used advanced methods such as restricted cubic splines to carefully examine the shape of the associations of total haemoglobin with mortality risks. Indeed, assuming the linearity of the continuous predictor-outcome association without further investigation, can lead to incorrect interpretation of the effects of the predictor on the outcome, particularly when the underlying relationship is not linear [34]. Systematically testing the significance of simple predictor transformations (restricted cubic splines for instance) has been advocated as a mean for exploring non-linearity [34].

### Conclusions and Perspectives

In conclusion, anaemia is a determinant of all-cause and cardiovascular mortality. Diabetic individuals with anaemia but no prior CVD have risks of mortality similar to those among CVD survivors with diabetes, but no anaemia. The previous focus on EPO for anaemia correction still leaves unaddressed other determinants of chronic anaemia in diabetes. Addressing these determinants and non-optimal cardiovascular risk profile may improve the outcomes of patients. To be effective however, interventions should be implemented early in routine diabetes care, and not at the kidney impairment stage [2]. Anaemia can affect the interpretation of values of HbA1c, the standard test for metabolic control monitoring in diabetes. Systematic screening for anaemia would help identifying a subgroup of highly vulnerable diabetic patients whose outcomes may potentially be modified. Currently, people with diabetes are routinely screened for cardiovascular disease [35], not for anaemia while both, based on our findings convey similar mortality risk.

## Supporting Information

Figure S1 **Derivation of the analytic sample.**
(TIF)

Figure S2 **Hazard ratio and 95% confidence interval across fifths of total haemoglobin, for the association with cardiovascular disease (left column) and all-cause (right column) mortality.** Within each fifth, estimates (hazard ratios) are shown for the total cohort (black diamonds) and separately for participants without diabetes (black boxes) and those with diabetes (black plain triangle). The vertical bars about the hazard ratios (broken for those with diabetes) represent the 95% confidence interval. Arrow-heads indicate that the 95% confidence interval bars have been truncated. For each outcome, figures are shown for the total cohort (upper panels), and separately for men (middle panels) and women (lower panels).
(TIF)

Table S1 Profile of participants included and those excluded.
(DOC)

Table S2 Baseline characteristics by status for anaemia and existing cardiovascular disease (CVD) in participants with and without diabetes.
(DOC)

Table S3 Baseline characteristics across fifths of total haemoglobin according to diabetes status.
(DOC)

Table S4 Unadjusted Incidence of Cardiovascular and all-cause mortality per 1000 person-years of follow-up and adjusted hazard ratio by status for diabetes, anaemia and existing cardiovascular disease.
(DOC)

Table S5 Fit statistics for various coding of total haemoglobin in relation with all-cause and cardiovascular mortality risk.
(DOC)
