# Supplementary figures and images for: Anaemia, Haemoglobin Level and Cause-Specific Mortality in People with and without Diabetes
Source: PLoS One. 2012 Aug 2;7(8):e41875. doi: 10.1371/journal.pone.0041875 (PMC3410893; doi:10.1371/journal.pone.0041875)

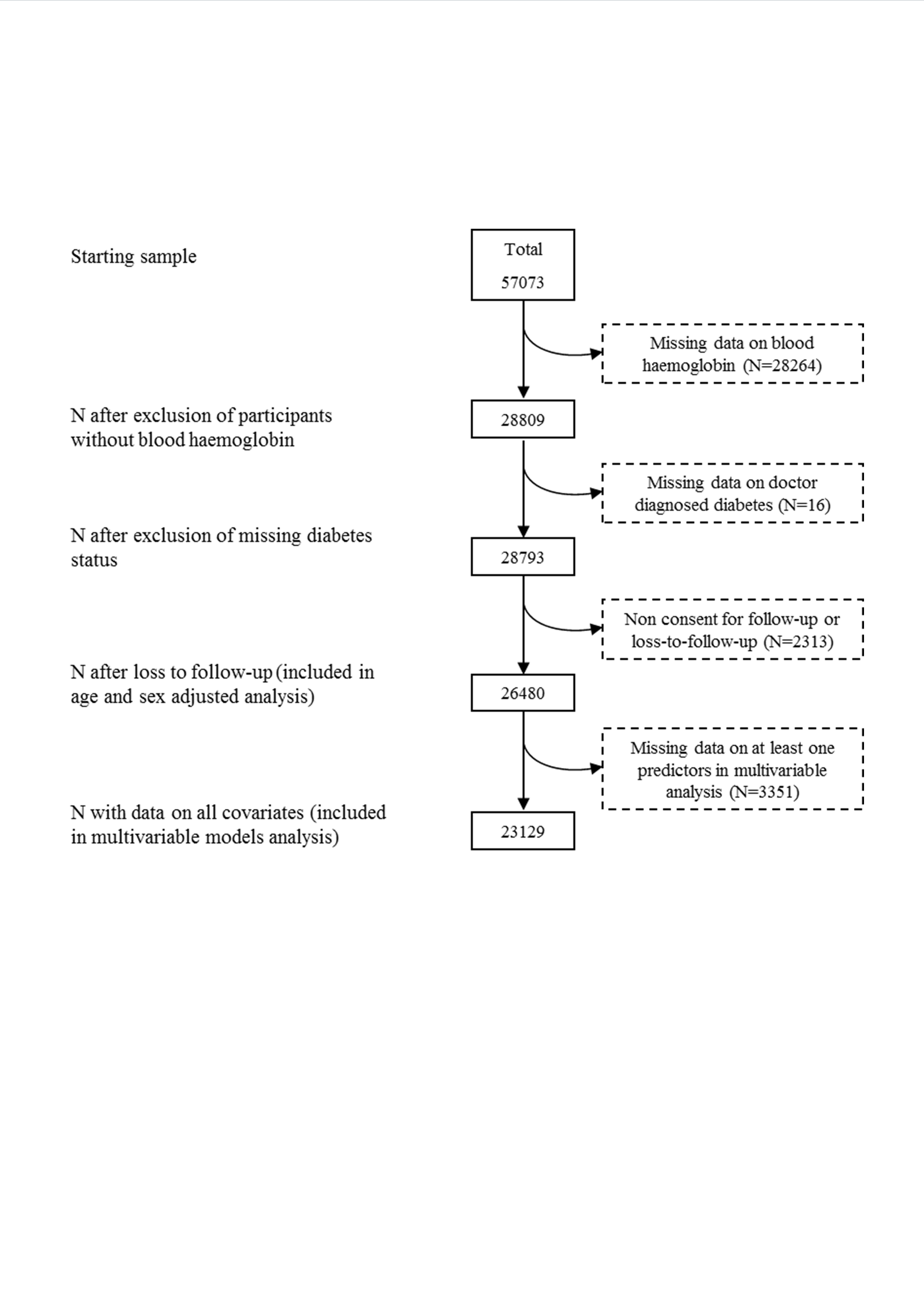

Supplement: Figure S1 — Derivation of the analytic sample. (TIF) [file pone.0041875.s001.tif]

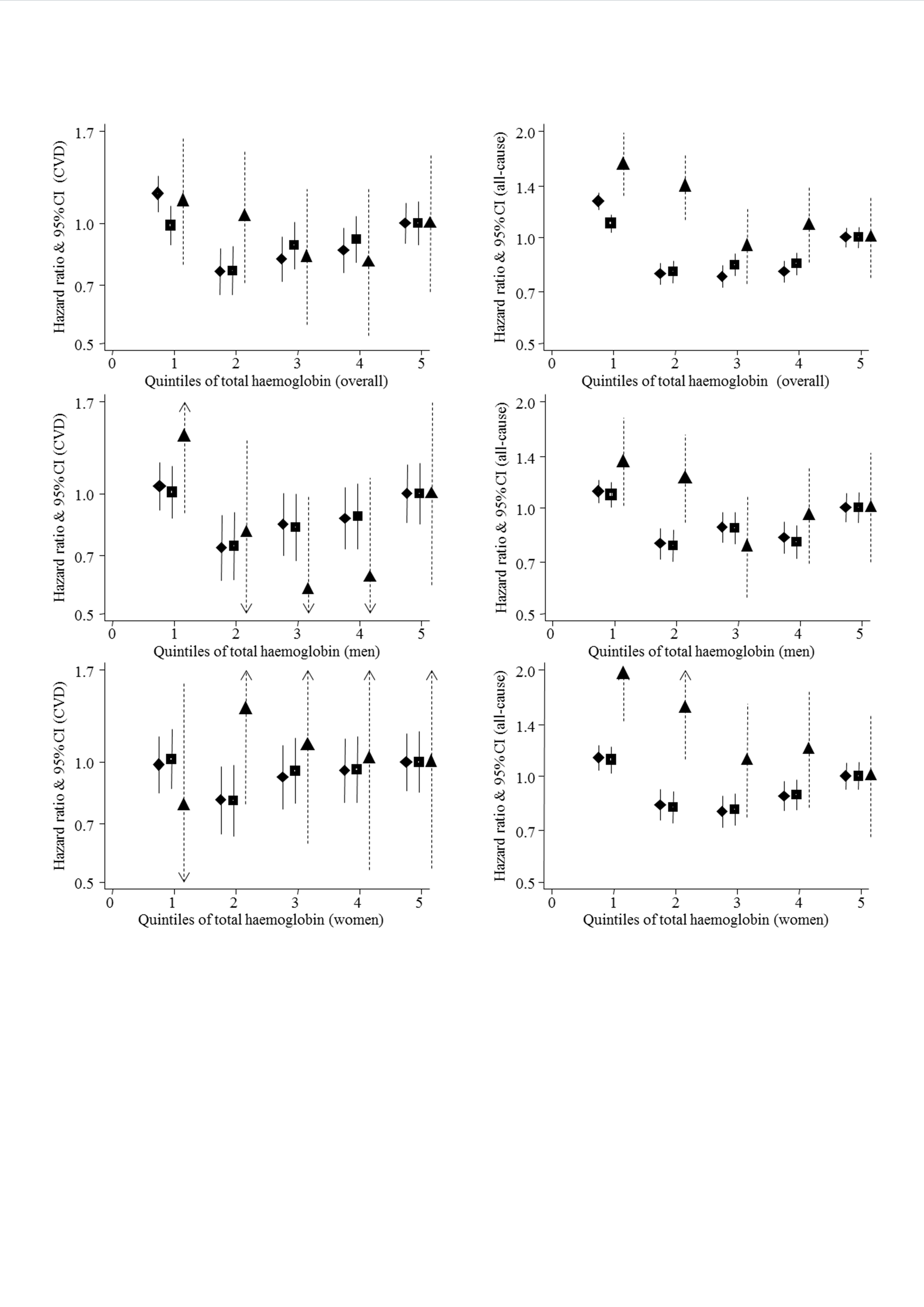

Supplement: Figure S2 — Hazard ratio and 95% confidence interval across fifths of total haemoglobin, for the association with cardiovascular disease (left column) and all-cause (right column) mortality. Within each fifth, estimates (hazard ratios) are shown for the total cohort (black diamonds) and separately for participants without diabetes (black boxes) and those with diabetes (black plain triangle). The vertical bars about the hazard ratios (broken for those with diabetes) represent the 95% confidence interval. Arrow-heads indicate that the 95% confidence interval bars have been truncated. For each outcome, figures are shown for the total cohort (upper panels), and separately for men (middle panels) and women (lower panels). (TIF) [file pone.0041875.s002.tif]
