# Supplementary material for: Anaemia, Haemoglobin Level and Cause-Specific Mortality in People with and without Diabetes
Source: PLoS One. 2012 Aug 2;7(8):e41875. doi: 10.1371/journal.pone.0041875 (PMC3410893; doi:10.1371/journal.pone.0041875)
Supplement: Table S1 — Profile of participants included and those excluded. (DOC) [file pone.0041875.s003.doc]

**Table S1** – Profile of participants included and those excluded

| **Variables** | **Participants in**  **the main analysis** | **Participants excluded** | **p-value** |
| --- | --- | --- | --- |
| N | 26,480 | 30,593 |  |
| Women, n (%) | 14,345 (54.2%) | 27,805 (58.2%) | <0.001 |
| Mean age, years (SD) | 55.0 (13.6) | 57.7 (15.8) | <0.001 |
| Current smoking, n (%) | 6958 (26.3%) | 7079 (23.7%) | <0.001 |
| Mean systolic blood pressure, mmHg (SD) | 137 (20) | 137 (21) | 0.65 |
| Mean body mass index, kg/m^2^ (SD) | 26.9 (4.6) | 27.3 (4.6) | <0.001 |
| Mean waist circumference, cm (SD) | 90 (13) | 91 (13) | <0.001 |
| Mean waist/hip ratio | 0.86 (0.09) | 0.87 (0.09) | 0.002 |
| Mean total cholesterol, mmol/l (SD) | 5.9 (1.2) | 5.8 (1.1) | <0.001 |
| Median CRP, mg/dl (25^th^-75^th^ percentiles) | 1.8 (0.8-4.0) | 1.3 (0.6-3.0) | <0.001 |

SD, standard deviation
