# Supplementary material for: Anaemia, Haemoglobin Level and Cause-Specific Mortality in People with and without Diabetes
Source: PLoS One. 2012 Aug 2;7(8):e41875. doi: 10.1371/journal.pone.0041875 (PMC3410893; doi:10.1371/journal.pone.0041875)
Supplement: Table S2 — Baseline characteristics by status for anaemia and existing cardiovascular disease (CVD) in participants with and without diabetes. (DOC) [file pone.0041875.s004.doc]

**Table S2 - Baseline characteristics by status for anaemia and existing cardiovascular disease (CVD) in participants with and without diabetes**

|  | No diabetes | | | | |  | Diabetes | | | | |
| --- | --- | --- | --- | --- | --- | --- | --- | --- | --- | --- | --- |
| Anaemia | No | Yes | No | Yes | p-value* |  | No | Yes | No | Yes | p-value* |
| Existing CVD | No | No | Yes | Yes |  |  | No | No | Yes | Yes |  |
| N | 21396 | 1793 | 2046 | 299 |  |  | 595 | 93 | 216 | 42 |  |
| Women (%) | 53.9 | 73.1 | 45.6 | 51.2 | <0.001 |  | 46.5 | 51.6 | 37.0 | 45.2 | 0.05 |
| Mean age, year (SD) | 53 (13) | 57 (17) | 66 (11) | 73 (13) | <0.001 |  | 61 (12) | 68 (15) | 65 (9) | 71 (12) | <0.001 |
| Current smoking (%) | 27.6 | 15.2 | 26.0 | 14.8 | <0.001 |  | 23.9 | 13.0 | 25.5 | 9.5 | 0.008 |
| Mean systolic blood pressure, mmHg (SD) | 136 (20) | 134 (21) | 144 (22) | 142 (25) | <0.001 |  | 146 (22) | 146 (27) | 146 (22) | 143 (21) | 0.81 |
| Resting heart rate, bpm (SD) | 71 (11) | 71 (11) | 69 (12) | 71 (13) | <0.001 |  | 75 (12) | 75 (10) | 70 (13) | 73 (10) | <0.001 |
| Mean body mass index, kg/m^2^ (SD) | 27 (4) | 26 (5) | 28 (5) | 26 (5) | <0.001 |  | 29 (5) | 27 (4) | 30 (5) | 27 (4) | <0.001 |
| Mean waist circumference, cm (SD) | 90 (13) | 85 (12) | 95 (13) | 91 (14) | <0.001 |  | 98 (13) | 94 (10) | 101 (12) | 94 (12) | <0.001 |
| Mean waist/hip ratio (SD) | 0.86 (0.08) | 0.83 (0.08) | 0.90 (0.09) | 0.89 (0.08) | <0.001 |  | 0.91 (0.08) | 0.90 (0.06) | 0.94 (0.08) | 0.92 (0.08) | <0.001 |
| Mean total cholesterol, mmol/l (SD) | 5.9 (1.2) | 5.4 (1.1) | 6.2 (1.3) | 5.6 (1.2) | <0.001 |  | 5.9 (1.2) | 5.2 (1.2) | 5.8 (1.0) | 5.3 (1.2) | <0.001 |
| Mean haemoglobin, mg/dl (SD) | 14.2 (1.2) | 11.4 (1.0) | 14.3 (1.2) | 11.5 (1.0) | <0.001 |  | 14.4 (1.2) | 11.5 (1.2) | 14.5 (1.2) | 11.5 (1.0) | <0.001 |
| Median CRP, ml/l (25^th^-75^th^ percentiles) | 1.7 (0.7-3.7) | 1.5 (0.6-4.0) | 2.9 (1.3-5.7) | 3.6 (1.2-7.3) | <0.001 |  | 2.9 (1.4-5.8) | 3.5 (0.9-16.6) | 3.7 (1.7-9.0) | 3.6 (1.3-6.7) | <0.001 |

***** p-values are for the differences across the 4 subgroups and are derived from analysis of the variance (ANOVA) for continuous variables, and from chi square for categorical variables
