# Supplementary material for: Anaemia, Haemoglobin Level and Cause-Specific Mortality in People with and without Diabetes
Source: PLoS One. 2012 Aug 2;7(8):e41875. doi: 10.1371/journal.pone.0041875 (PMC3410893; doi:10.1371/journal.pone.0041875)
Supplement: Table S3 — Baseline characteristics across fifths of total haemoglobin according to diabetes status. (DOC) [file pone.0041875.s005.doc]

**Table S3 – Baseline characteristics across fifths of total haemoglobin according to** diabetes status

| Variables | No diabetes | | | | | |  | Diabetes | | | | | | p-value |
| --- | --- | --- | --- | --- | --- | --- | --- | --- | --- | --- | --- | --- | --- | --- |
| Fifths of haemoglobin | Q1 | Q2 | Q3 | Q4 | Q5 | p-trend |  | Q1 | Q2 | Q3 | Q4 | Q5 | p-trend |  |
| N | 5340 | 4952 | 5147 | 5186 | 5009 |  |  | 185 | 192 | 192 | 186 | 191 |  |  |
| Median haemoglobin  (min-max), g/dl | 12.3  (4.8-13.9) | 13.0  (12.5-14.5) | 13.5  (13.1-15.0) | 14.1  (13.6-15.6) | 15.7  (14.2-20.1) |  |  | 12.0  (7.5-13.4) | 13.5  (12.3-14.3) | 14.4  (13.1-14.9) | 15.1  13.8-15.7) | 15.9  (14.6-17.9) |  |  |
| Age (years) | 56.6 (15.6) | 54.2 (13.4) | 53.7 (13.0) | 53.9 (12.8) | 55.2 (12.7) | <0.001 |  | 67.8 (13.7) | 63.2 (12.8) | 62.0 (12.6) | 61.4 (10.4) | 60.2) | <0.001 | <0.001 |
| Women (%) | 55.1 | 55.4 | 55.1 | 53.6 | 53.4 | 0.02 |  | 44.9 | 46.3 | 44.8 | 40.9 | 47.1 | 0.08 | <0.001 |
| Current smoking (%) | 17.4 | 20.6 | 24.0 | 29.4 | 41.0 | <0.001 |  | 10.9 | 17.7 | 18.2 | 26.9 | 38.7 | <0.001 | 0.008 |
| Prior CVD (%) | 12.4 | 8.0 | 7.4 | 7.9 | 10.1 | <0.001 |  | 30.8 | 27.1 | 29.2 | 21 | 28.3 | 0.29 | <0.001 |
| Systolic blood pressure (mm Hg) | 134.2 (20.5) | 134.9 (19.6) | 135.9 (19.9) | 137.9 (19.5) | 141.2 (20.6) | <0.001 |  | 145.0 (23.7) | 147.2 (23.8) | 143.8 (22.9) | 145.3 (20.8) | 147.6 (20.3) | 0.54 | <0.001 |
| Resting heart rate (bpm) | 70 (11) | 69 (11) | 70 (11) | 71 (11) | 73 (12) | <0.001 |  | 73 (11) | 73 (11) | 72 (12) | 75 (13) | 76 (13) | 0.01 | <0.001 |
| Body mass index (kg/m^2^) | 26 (4.5) | 26.4 (4.3) | 26.8 (4.4) | 27.4 (4.6) | 27.7 (4.8) | <0.001 |  | 27.4 (4.4) | 28.5 (4.8) | 28.8 (4.8) | 29.4 (4.9) | 29.9 (5.9) | <0.001 | <0.001 |
| Waist circumference (cm) | 87.5 (12.6) | 88.3 (12.6) | 89.3 (12.5) | 90.9 (13) | 92.3 (13.2) | <0.001 |  | 95.7 (12.1) | 97.9 (13.3) | 98.3 (13.1) | 98.7 (12.5) | 99.5 (13.3) | 0.008 | <0.001 |
| Waist/hip ratio | 0.85 (0.09) | 0.85 (0.09) | 0.86 (0.08) | 0.87 (0.09) | 0.88 (0.09) | <0.001 |  | 0.91 (0.07) | 0.91 (0.08) | 0.92 (0.09) | 0.92 (0.08) | 0.93 (0.08) | 0.03 | <0.001 |
| Total cholesterol (mmol/l) | 5.6 (1.2) | 5.8 (1.1) | 5.9 (1.1) | 6.1 (1.2) | 6.2 (1.2) | <0.001 |  | 5.3 (1.1) | 5.8 (1.2) | 5.7 (1.0) | 6.0 (1.0) | 6.0 (1.3) | <0.001 | <0.001 |
| Median CRP mg/l | 1.6 (0.6-4.1) | 1.7 (0.7-3.8) | 1.9 (0.9-4.0) | 1.7 (0.8-3.8) | 1.9 (0.9-4.0) | <0.001 |  | 4.2 (1.0-14.2) | 3.3 (1.1-6.5) | 3.4 (1.4-7.3) | 3.0 (1.5-6.0) | 2.7 (1.6-5.3) | <0.001 | <0.001 |

CRP, c-reactive protein; CVD, cardiovascular disease; p-trend, p for linear trend; p-value, p for difference between participants with diabetes and those without.
