# Supplementary material for: Anaemia, Haemoglobin Level and Cause-Specific Mortality in People with and without Diabetes
Source: PLoS One. 2012 Aug 2;7(8):e41875. doi: 10.1371/journal.pone.0041875 (PMC3410893; doi:10.1371/journal.pone.0041875)
Supplement: Table S4 — Unadjusted Incidence of Cardiovascular and all-cause mortality per 1000 person-years of follow-up and adjusted hazard ratio by status for diabetes, anaemia and existing cardiovascular disease. (DOC) [file pone.0041875.s006.doc]

**Table S4 - Unadjusted Incidence of Cardiovascular and all-cause mortality per 1000 person-years of follow-up and adjusted hazard ratio by status for diabetes, anaemia and existing cardiovascular disease**

| **Baseline classification** | | |  | **CVD mortality** | |  |  | **All-cause mortality** | | |
| --- | --- | --- | --- | --- | --- | --- | --- | --- | --- | --- |
| Existing CVD | Anaemia | Diabetes |  | Event rate (/1000 prs) | Hazard ratio | Hazard ratio* |  | Event rate (/1000 prs) | Hazard ratio | Hazard ratio* |
| No | No | No |  | 3.05 (2.83-3.29) | 1 (Reference) | 1 (Reference) |  | 12.40 (11.95-12.87) | 1 (Reference) | 1 (Reference) |
| No | Yes | No |  | 7.03 (5.87-8.41) | 1.78 (1.40-2.26) | 2.30 (1.55-3.42) |  | 26.63 (24.28-29.21) | 1.49 (1.32-1.69) | 1.61 (1.25-2.10) |
| No | No | Yes |  | 11.74 (9.19-14.99) | 2.16 (1.59-2.95) | 1.69 (1.22-2.36) |  | 34.84 (30.22-40.17) | 1.66 (1.39-1.97) | 1.29 (1.07-1.57); |
| Yes | No | No |  | 18.90 (16.98-21.03) | 2.38 (2.02-2.81) | 2.50 (2.01-3.11) |  | 45.68 (42.64-48.93) | 1.60 (1.46-1.76) | 1.59 (1.38-1.83); |
| No | Yes | Yes |  | 15.37 (8.27-28.57) | 1.71 (0.76-3.85) | 1.42 (0.45-4.46) |  | 70.71 (52.96-94.40) | 2.13 (1.48-3.07) | 1.80 (1.03-3.13); |
| Yes | No | Yes |  | 26.34 (19.60-35.39) | 4.17 (2.93-5.92) | 4.64 (3.08-7.00) |  | 64.65 (53.54-78.07) | 2.68 (2.14-3.36) | 2.70 (2.01-3.62); |
| Yes | Yes | No |  | 28.92 (22.21-37.67) | 2.51 (1.70-3.72) | 2.40 (1.45-3.97) |  | 93.08 (80.33-107.85) | 2.14 (1.73-2.66) | 1.75 (1.27-2.40); |
| Yes | Yes | Yes |  | 70.92 (42.75-117.64) | 5.34 (2.38-12.00) | - |  | 151.30 (106.99-213.95) | 3.25 (1.88-5.62) | 2.70 (1.39-5.23) |

Cox models are stratified by cohort and adjusted for age, sex, smoking systolic blood pressure, total cholesterol, body mass index

* Model are further adjusted for CRP in the subgroup of 13,228 participants with data available on CRP levels at baseline. The small number of them who had both anaemia, diabetes and history of CVD at baseline resulted in no outcome of CVD death recorded during follow-up
