# Supplementary material for: Anaemia, Haemoglobin Level and Cause-Specific Mortality in People with and without Diabetes
Source: PLoS One. 2012 Aug 2;7(8):e41875. doi: 10.1371/journal.pone.0041875 (PMC3410893; doi:10.1371/journal.pone.0041875)
Supplement: Table S5 — Fit statistics for various coding of total haemoglobin in relation with all-cause and cardiovascular mortality risk. (DOC) [file pone.0041875.s007.doc]

**Table S5** – Fit statistics for various coding of total haemoglobin in relation with all-cause and cardiovascular mortality risk

| Coding for haemoglobin |  | **All-Cause mortality** | |  | **CVD mortality** | |
| --- | --- | --- | --- | --- | --- | --- |
|  | df | LR | AIC |  | LR | AIC |
| None* | 2 | 5827.16 | 71597.23 |  | 1980.77 | 20300.06 |
| Linear | 3 | 5843.37 | 71583.03 |  | 1980.91 | 20301.92 |
| RCS 3 knots | 4 | 5909.08 | 71519.31 |  | 1993.69 | 20291.14 |
| RCS 4 knots | 5 | 5912.68 | 71517.71 |  | 1993.90 | 20292.94 |
| Polynomial 2^nd^ order | 4 | 5895.20 | 71533.19 |  | 1990.34 | 20294.49 |
| Polynomial 3^rd^ order | 5 | 5919.98 | 71510.41 |  | 1991.21 | 20295.62 |
| Tertiles | 4 | 5838.13 | 71606.65 |  | 1982.96 | 20303.73 |
| Quartiles | 5 | 5862.87 | 71583.91 |  | 1989.64 | 20299.06 |
| Quintiles | 6 | 5877.43 | 71571.34 |  | 1989.31 | 20301.39 |

* Model with covariates (sex and age) only

AIC, Akaike’s information criterion; df, degree of freedom of the model; LR, likelihood ratio chi, square; RCS, restricted cubic splines
